# Challenges in the Diagnosis and Treatment of Oral Amelanotic Malignant Melanoma: A Case Report

**DOI:** 10.7759/cureus.57875

**Published:** 2024-04-08

**Authors:** Oana A Rosu, Madalina I Tolea, Andreea I Parosanu, Miruna I Stanciu, Horia T Cotan, Cornelia Nitipir

**Affiliations:** 1 Oncology, Elias Emergency University Hospital, Bucuresti, ROU; 2 Oncology, Elias Emergency University Hospital, Bucharest, ROU; 3 Oncology, Carol Davila University of Medicine and Pharmacy, Bucharest, ROU; 4 Medical Oncology, Elias Emergency University Hospital, Bucharest, ROU

**Keywords:** pathology, immunotherapy, melanoma, amelanotic, oral

## Abstract

Oral malignant melanoma (OMM) is extremely rare and usually has a poor prognosis. Early diagnosis is very important and can improve survival but it is usually difficult due to a lack of symptomatology. We present the first case of a 39-year-old East European woman with oral amelanotic melanoma, who underwent surgery and adjuvant immunotherapy; however, after six months, she developed local recurrence. The patient continued immunotherapy with external radiotherapy targeting the oral tumor recurrence. During therapy, imagistic reevaluation brought evidence of bones, lungs, liver, endotracheal, and brain metastases. Histological differential diagnosis between amelanotic OMM and leiomyosarcoma was necessary to establish the right course of treatment. A series of complications further delayed chemotherapy administration, making the treatment in this case very challenging. The patient had a significant, although late response to immunotherapy, and maintained a good performance status during disease progression with a survival of 15 months until present.

## Introduction

Oral malignant melanoma (OMM) is a very rare and aggressive disease accounting for only 0.5% of all the malignancies located in the oral region and represents under 1% of all melanomas [1]. The overall five-year survival rate of this disease is between 7% and 40% [2]. Although there is no exact treatment modality well established, studies showed that early detection and treatment with surgery and radiotherapy improve OMM general survival [3]. This particular rare site raises many challenges for the course of treatment. Naganawa et al. observed that seven out of 19 patients with OMM developed osteoradionecrosis grade 2 or 3 after radiotherapy. One of the most important risk factors for developing this complication was the presence of teeth within the planning target volume [2]. Alcohol intake, cigarette smoking, and oral or dental lesions represent other risk factors described in the literature involved in the development of OMM [4]. Despite its rare occurrence, there is evidence to suggest OMM tends to metastasize early in the regional lymph nodes, while the two most common distance metastasis sites are represented by the lung and liver [5]. We will discuss the case of an advanced amelanotic OMM with local recurrence and multiple metastatic sites: disease involvement in the lymphatic nodules, endotracheal tissue, lung, liver, bones, and brain, and the challenges we encounter during treatment. In addition, we will discuss some of the existing literature related to this topic.

## Case presentation

A 39-year-old Caucasian woman, with no family history of cancer, no personal history of malignancy or comorbidities presented to another unit for a voluminous gingival mass located on the right side of the mandible, corresponding to the alveolar site of the eighth tooth. There is no history of alcohol consumption, substance abuse, smoking cigarettes or other tobacco products, dental issues, or chewing habits, as well as no locoregional trauma before the diagnosis or development of the tumor in this case.

Soon after the presentation, the patient underwent an incisional biopsy, and the histopathological examination stated the diagnosis of oral amelanotic mucosal melanoma but with a slight possibility for sarcoma differentiation. Further, there were conducted immunohistochemical (IHC) tests, which revealed positive staining of SOX10 (SRY-related HMG-box gene 10) and S100 (small EF-hand calcium-binding proteins) in the tumor cells, while AE1/AE3 (a mix of two different clones of cytokeratin monoclonal antibody) led to negative reactions in the tumor tissue. IHC tests were incomplete, as Melan A and HMB45 were not tested. To determine the anatomic extent of the tumor, the patient underwent a computer tomography (CT) scan of the head and neck. To rule out metastatic disease, cerebral magnetic resonance imaging (MRI), and positron emission tomography (PET) were also conducted. Imaging results described a 3-3.5 cm diameter tumor on the right side of the mandible and metabolically active right submandibular lymph nodes.

Wide excision of the primary tumor and right neck lymph node dissection were consequently performed. The histopathological examination described a 2.5 cm diameter ulcerated tumor with 11 mm depth of invasion, and metastatic involvement in a single 2 cm ipsilateral lymph node, confirming the diagnosis of stage III (TNM staging: pT3 pN1) amelanotic OMM. IHC tests revealed positive SOX10 and S100 markers and negative Melan A (melanoma antigen recognized by T-cells) and HMB45 (human melanoma black monoclonal antibody). Moreover, the BRAF (v-RAF murine sarcoma viral oncogene homolog B1) gene testing was negative.

The tumor board from the previous unit where the patient was treated decided to initiate systemic therapy with Pembrolizumab 200 mg every three weeks.

**Figure 1:**
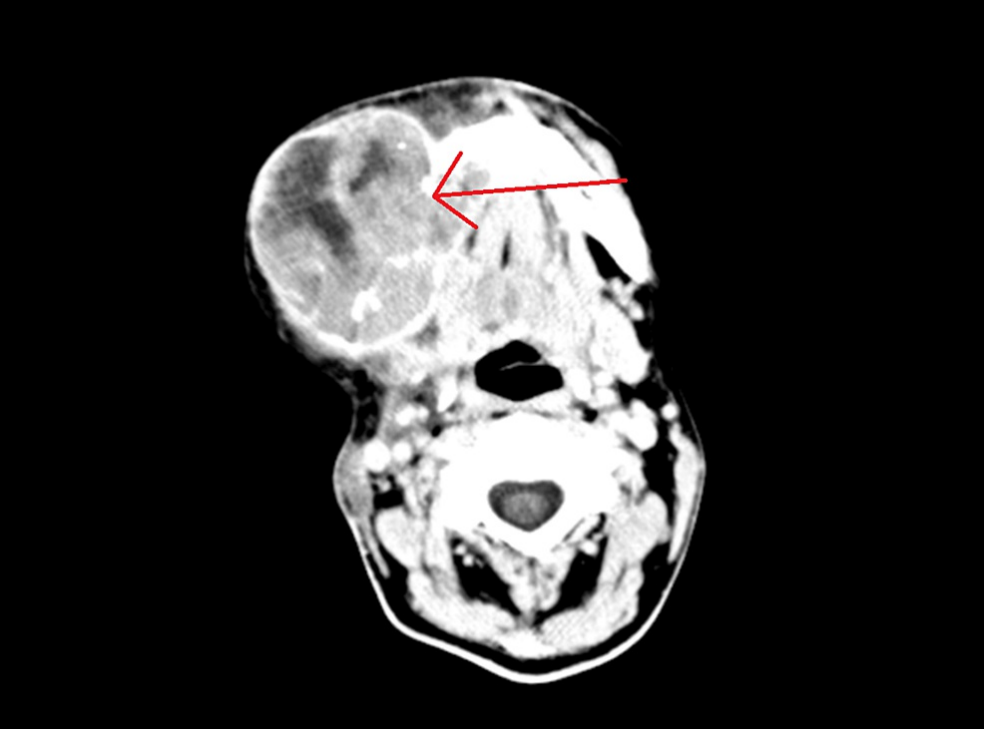
Cerebral MRI axial view. Magnetic resonance imaging (MRI) representation of the right mandibular deep infiltrative tumor recurrence measuring 93 mm anteroposterior diameter, 55 mm laterolateral diameter, and 100 mm craniocaudal diameter, exhibiting inhomogeneous gadolinophilia and central necrosis. The tumor is invasive, extending medially into the oral floor adjacent to the pharyngeal Rossenmuller recess, posteriorly reaching the carotid lodge and causing displacement, and cranially contacting the lateral wall of the right maxillary sinus (zygomatic complex). Anteriorly, it extends to the mandibular chin, enveloping the mandibular lateral incisors. Additionally, it invades the masseter and right pterygoid muscles, as well as the superficial subcutaneous tissue.

Due to the delicate political situation in their country, after six months of immunotherapy with Pembrolizumab there, the patient presented to our Oncological Department at Elias Emergency Hospital, Romania, to continue treatment and investigations. Clinical examination revealed a large painful mass on the right side of the mandible, measuring around 6-7 cm in diameter (Figure 1).

Cerebral and cervical MRI and computed tomography of the thorax, abdomen, and pelvis were performed as part of the initial evaluation (Figure 1). Results showed local recurrence of the primary tumor, which invaded the oral muscular tissue and the pterygoid-facial region. 

We decided to perform an incisional biopsy of the local recurrence and BRAF gene retesting. The histopathological report described a fragment of squamous mucosal tissue showing reactive changes. At the level of a group resection margin, medium and large epithelioid cells were observed, exhibiting a unitary or supraunitary nucleus/cytoplasm ratio; eosinophilic cytoplasm with a frosted glass appearance, round-oval nucleus, or a polycyclic outline; homogeneous chromatin; and one or two prominent nucleoli. Some cells displayed highlighting mitotic figures. In the rest of the examined tissue, the subepithelial stroma showed focal minimal to moderate lymphoplasmacytic inflammatory infiltrate, occasionally exocytic, with rare exocytic neutrophils. Corroborated with the medical history of the patient and the clinical presentation, this report was consistent with OMM recurrence. In addition, IHC tests were conducted on this material. The results confirmed the diagnosis of OMM recurrence with positive markers such as S100, SOX10, Melan A, and HMB45. Additionally, the test results were negative again for the BRAF mutation upon retesting.

Therefore, the multidisciplinary tumor board decided that the tumor recurrence was unresectable and recommended palliative external radiotherapy (IMRT-VMAT= intensity-modulated radiotherapy - volumetric modulated arc radiotherapy), with a total dose of 39 Gy/tumoral volume and systemic therapy with Nivolumab and Ipilimumab for four cycles followed by monotherapy with Nivolumab.

**Figure 2:**
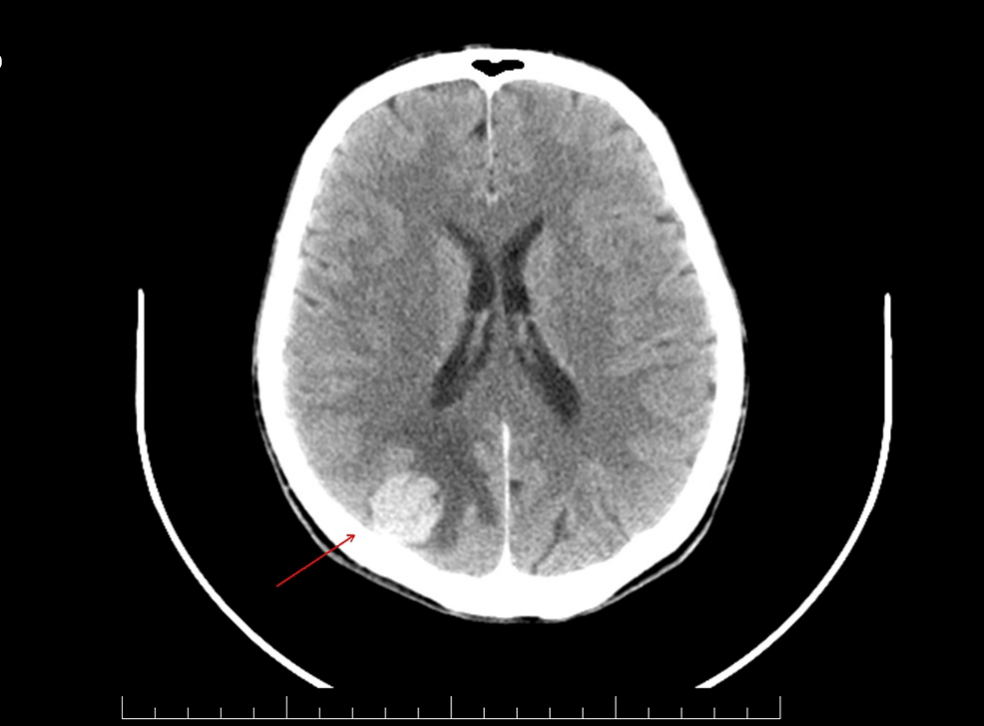
Cerebral MRI axial view. In the occipital area of the right main hemisphere, a suspicious lesion with a signal can be visualized on magnetic resonance imaging. It appears moderately increased on T2 and fluid-attenuated inversion recovery (FLAIR) sequences, isotensive on T1, and narrow on diffusion-weighted imaging (DWI), measuring 27 mm anteroposterior diameter, 22 mm laterolateral diameter, and 23 mm craniocaudal diameter. Additionally, a pathological contrast accumulation area of 2 mm in diameter is observed in the cortico-subcortical area of the main cerebral hemisphere, suggesting the appearance of brain metastasis.

Two months later, the patient presented with a severe headache, and the cerebral MRI showed a secondary brain tumor in the right parietal lobe (Figure 2). The multidisciplinary team, including a neurosurgeon and a radiotherapist, recommended Gamma Knife surgery with a total dosage of 2.3 Gy (3 J) for brain metastasis.

**Figure 3:**
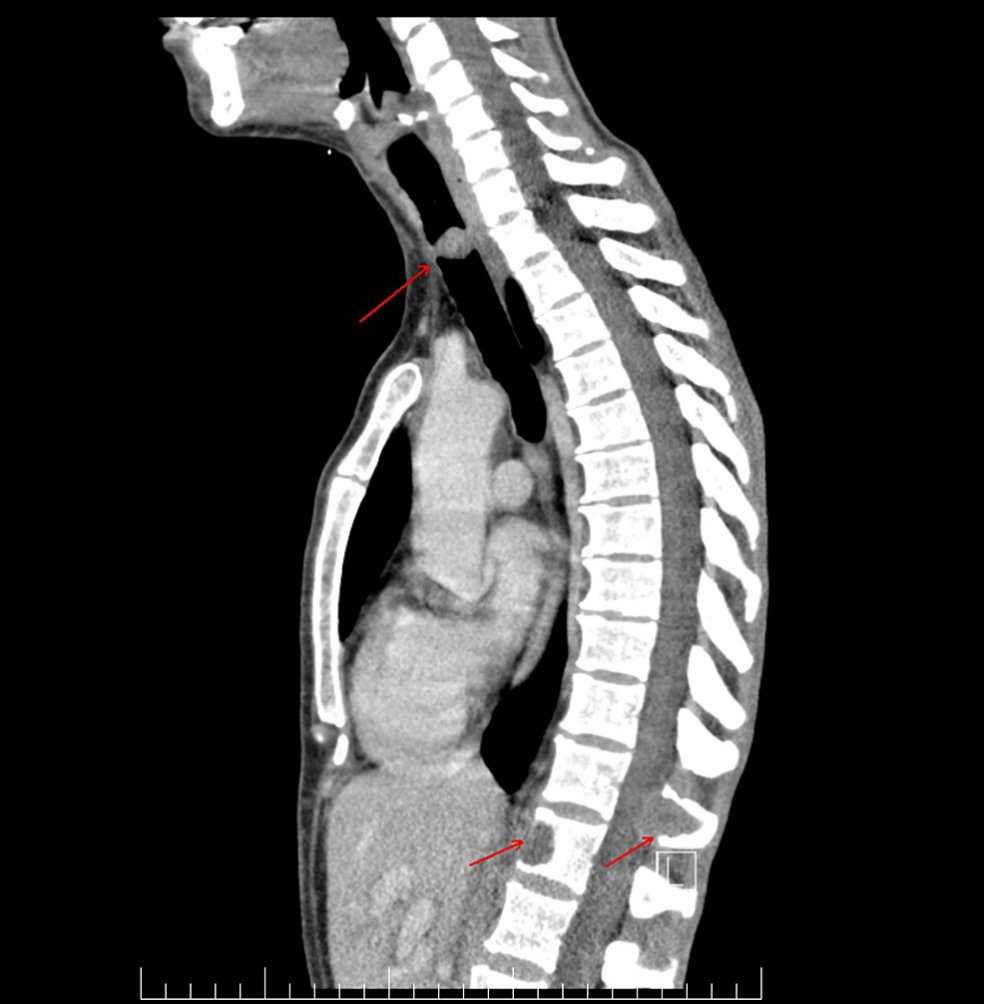
Sagittal view of the spinal cord CT. A vegetative round intraluminal tracheal lesion measuring 15 mm in the anteroposterior diameter, 15 mm in laterolateral diameter, and 13 mm in craniocaudal diameter, creating a quasi-complete obstruction of the tracheal lumen. This lesion is situated at the level of the cervical trachea, specifically at the C7-T1 vertebrae. An osteolytic lesion is observed at the level of the posterior arch of the T11 vertebral body, measuring 30 mm in anteroposterior diameter, 26 mm in laterolateral diameter, and 29 mm in craniocaudal diameter. It invades the posterior epidural space, demonstrates neuroforaminal invasion, and results in vertebral canal stenosis at this level (anteroposterior diameter = 70 mm, compared to 13 mm above and below). Another osteolytic lesion is described in the 1/3 anterior part of the T12 vertebral body, measuring 13 mm in anteroposterior diameter and 21 mm in craniocaudal diameter.

The patient rapidly developed severe dyspnea, and an emergency bronchoscopy revealed an endotracheal tumor fixed on the posterior wall, partially obstructing the airflow passage at the level of the third tracheal ring (Figure 3). Tracheostomy was not an option because the tumor infiltrated the mediastinum. Therefore, an electrosurgical resection was conducted. The histopathological examination described a tumor proliferation with a sarcomatous appearance, featuring fusiform cells arranged in bundles and exhibiting atypical mitoses. Further immunohistochemistry (IHC) tests revealed negativity in tumor cells for AE1/AE3, CK7, and CK20, while S100, vimentin, and SMA were positive. Additionally, CD34 was positive in vessels but negative in tumor cells. These results raised the suspicion of a new primary tumor development and was diagnosed as a leiomyosarcoma based on these findings.

**Figure 4:**
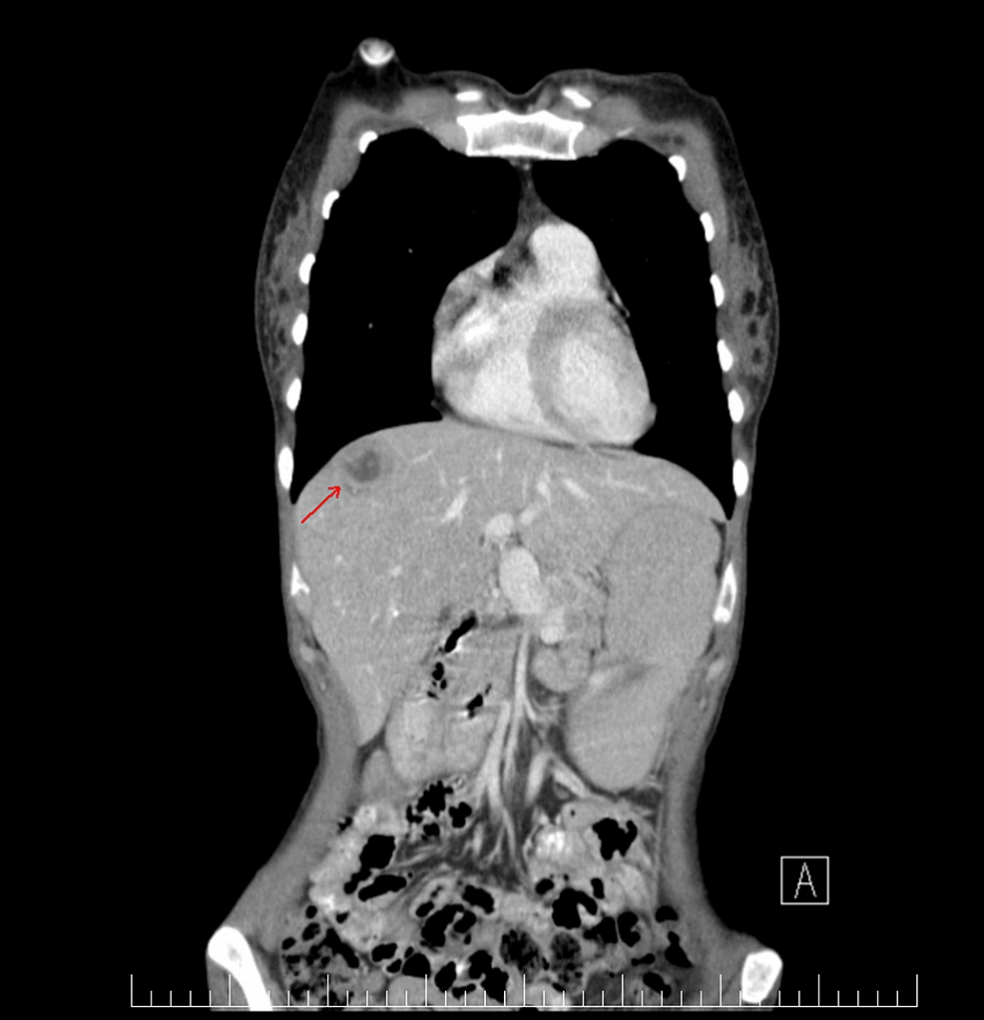
Coronal view of thoraco-abdomino-pelvic CT scan. The liver has normal dimensions and regular contours. Within the VIII segment, there is a hypodense lesion that is well-delimited, with axial diameters of approximately 51 mm in anteroposterior diameter and 45 mm in laterolateral diameter. This lesion exhibits spontaneous iso/hyposignal and is non-encapsulated. During the arterial phase, there is early enhancement, except for a fibrotic central area, and a tendency toward homogenization is observed in the late sequences. Additionally, areas of hypervascularization are noted.

**Figure 5:**
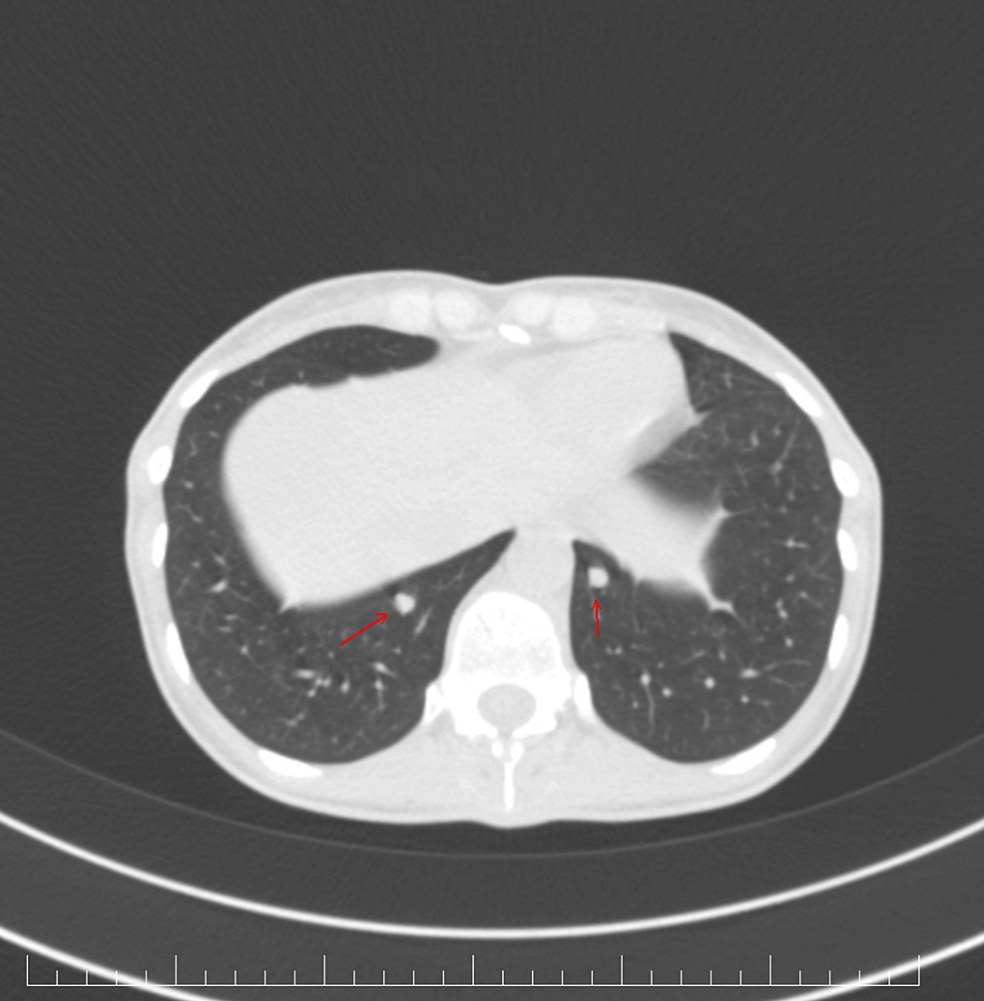
Axial view of the thoracic CT scan. Multiple pulmonary intraparenchymal nodular tissue lesions are observed, iodophile and well delimited, distributed bilaterally, predominantly at the basal regions. These lesions have a maximum diameter of 6.6 mm, noted at the level of the right Fowler segment, exhibiting suggestive features of secondary determinations.

We also performed a whole-body CT scan that revealed multiple liver, lung, and bone metastases (Figures 3-5). For symptomatic bone metastases, the patient also received palliative radiotherapy on the T11 and T12 vertebrae lesions, up to a total dosage of 30 Gy.

Concerns were raised about the accuracy of the histological results. To improve the accuracy of the diagnosis, we sent all the previous biopsies and surgical tissue samples for a second opinion in an experienced center. As these second opinion evaluations usually take more than 14 days to receive an extensive response, we decided to continue the patient's treatment with the information available at this point.

In consequence, the tumor board decided to start second-line Dacarbazine every three weeks and bisphosphonates (zoledronic acid) after consultation with a dental specialist.

Unfortunately, the chemotherapy was delayed because of another challenge. The patient was diagnosed with Streptococcus anginosus infection at the primary tumor site, which required 14 days of antibiotics. Moreover, after two cycles of therapy with Dacarbazine and zoledronic acid, the treatment was once again delayed because of a recurrent labial Herpes simplex virus infection, which needed antiviral medication. Finally, after another 14 days, the chemotherapy was safely reinitiated.

After eight weeks, the imagistic examination showed favorable results, with regression in brain metastasis after stereotactic radiotherapy, and a substantially decreased number of lung and liver metastases.

Meanwhile, the results from the second opinion experienced histopathological center became available to us but with a significant delay. The histopathological description of the examined tissue blocks from the biopsies of the mandibular tumor showed fragments of tumor proliferation composed of round and oval cells with eosinophilic cytoplasm, with enlarged nuclei of unequal volume, pleomorphic, with frequent mitoses, arranged in nests; tumor proliferation was covered by stratified squamous epithelium and infiltrates of striated muscle and seromucous glands, and the IHC test was positive on all blocks for Melan A, HMB45, S100, and SOX10. These final findings sustained the original diagnosis of amelanotic OMM for both the primary mandibular tumor and the recurrence.

The tracheal mass was histopathologically described as a tumor proliferation consisting of elongated fusiform cells with hyperchromic nuclei, exhibiting pleomorphism, bi- and multinucleated giant cells, and frequent mitoses. IHC tests revealed positivity for ACT, CD117, Calponin, Nestin, and Caldesmon in numerous tumor cells, while Desmin, CD34, ER, and DOG1 were negative. Additionally, ki67 staining showed 70% positivity. Based on these findings, the final diagnosis supported a mesenchymal proliferation consistent with gastrointestinal stromal tumor (GIST) with an increased mitotic rate. These findings contradict the original diagnosis of either a primary leiomyosarcoma or an amelanotic OMM metastasis.

The tumor board reunited again with these new data and decided to stop chemotherapy as the patient was facing new challenges. Right mandibular osteonecrosis occurred after four cycles of therapy with Dacarbazine and zoledronic acid. The patient was admitted to the Oncological Department and received antalgic and antibiotic therapies to provide symptomatic control and manage the local infection. After the resolution of pain and infection, the patient left against medical advice and was lost to follow-up. 

## Discussion

Primary OMM is an extremely rare disease accounting for 0.2%-8% of melanomas. It is mostly diagnosed in males over 60 years old, has a worse prognosis, and spreads faster than cutaneous malignant melanoma [6]. Even though the etiology of oral melanoma is poorly understood, the most common risk factors are trauma, alcohol, tobacco and smokeless tobacco products, and denture lesions or chewing habits [7].

Moreover, there are only a few cases of OMM described in the literature. Due to the lack of symptomatology and nonspecific radiological findings, most of the cases were diagnosed in advanced stages [6,8]. Over 75% of patients had regional lymph node involvement at diagnosis and up to 50% of patients presented with metastatic disease [8,9]. For example, Nisi et al. reported in an extensive review, including 447 cases of primary oral AMM, that more than 30% of patients were metastatic at the time of diagnosis [10]. In another review including 55 cases with oral amelanotic malignant melanoma (AMM), 23.6% of patients had tumors to the mandibular gingiva, 65.5% of patients were older than 68 years, and 43% were females [11].

Thus, our case report is even rarer because we presented a 39-year-old woman, with primary amelanotic melanoma of the mandibular gingiva and no previous risk factors.

Oral amelanotic melanoma has no clinical and radiological particularities. Therefore, histopathological examination is essential in providing an accurate and complete diagnosis. We faced challenges concerning diagnostic accuracy. Even if less than 2% of all melanomas lack pigment, up to two-thirds of oral melanomas are amelanotic [12]. AMM is defined as a tumor composed of non-pigmented melanocytes. However, in other reports, a tumor that lacks pigmentation clinically and has melanin pigmentation observed in the histopathological report is also included in this category [13]. Moreover, several literature findings highlight the challenges in the differential histopathological diagnosis of amelanotic melanoma and other diseases like oral scleritis [14], odontogenic neoplasm [15], clear cell sarcoma [16], and cases of oral sarcomas initially considered melanomas [17]. In our case, a differential diagnosis was made between amelanotic melanoma and a suspected leiomyosarcoma. Identifying malignant melanoma is generally straightforward when melanin pigment is present. However, the challenge arises with amelanotic melanomas because this subtype of melanoma can be a deceptive mimic, presenting a significant diagnostic difficulty. Primary mucosal melanoma is an uncommon neoplastic condition, constituting only 1.4% of all malignant melanomas. These melanomas that develop in mucosal tissues exhibit distinct pathobiology and clinical characteristics compared to cutaneous melanomas.

Additionally, mucosal melanomas display a varied histomorphology, encompassing small cells, spindle cells, pleomorphic cells, epithelioid cells, plasmacytoid cells, rhabdoid cells, undifferentiated cells, and mixed patterns [18]. An interesting particularity of this case is the refractory nature of melanoma to immunotherapy with a poor rate of response to both adjuvant Pembrolizumab and palliative Nivolumab + Ipilimumab and a favorable response to chemotherapy despite historical response rates of only 20% in patients treated with Dacarbazine [19]. The rapid progression after Pembrolizumab treatment is characteristic of immune checkpoint inhibitor refractory melanoma, which has an extremely poor prognosis. Moreover, during combined ICI treatment, the patient developed hyperprogression, which was defined by Ferrara et al. as RECIST progression at the first imaging evaluation [20]. This raises the question of a more frequent imaging evaluation during immunotherapy treatment, at least during the first 2-3 months.

We chose an IO-IO combination (Nivolumab+Ipilimumab) as a subsequent line of therapy despite progression on the previous anti-PD-1 antibody Pembrolizumab, based on the phase II trial SWOG 1616 [21]. In this trial, 92 patients with metastatic or unresectable melanoma who have progressed on single-agent PD-1 or PD-L1 and without previous treatment with CTLA-4 (cytotoxic T-lymphocyte-associated antigen 4) inhibitors were assigned to IO-IO combination of Nivolumab plus Ipilimumab or Ipilimumab monotherapy. At a follow-up of 25 months, the combination therapy improved progression-free survival (PFS; six-month PFS 34% vs. 13%, hazard ratio [HR] 0.94, 90% confidence interval [CI] 0.45-1.62). Objective responses were also higher (28% vs. 9%). Overall survival (OS) was similar (one-year OS 63% vs. 57% HR 0.94, 90% CI 0.45-1.62). A similar study by Friedman et al. [22] confirms the efficacy of Nivolumab plus Ipilimumab, although it was not superior to Ipilimumab monotherapy.

That being said, we believe that highly qualified pathologists helped us to attain a complete and accurate diagnosis but with a significant delay in the therapeutic process, as an expert’s second opinion usually takes some time to be obtained. This was necessary for our oncological team to understand the complex pathology of this particular patient. An extremely challenging therapeutic approach and the differential diagnosis was observed in this case of primary oral AMM and GIST, which was initially thought to be a primary leiomyosarcoma or suspected to be a secondary tumor from oral AMM.

Besides the complicated situation with the differential histopathological diagnosis, there were other unexpected problems along the therapeutic road in this case. 

Infection is one of the main causes of chemotherapy delay [23]. On the other hand, chemotherapy induces immunosuppression, which increases the risk of infection. 

In our case, the tumor necrosis caused secondary pyogenic infection with Streptococcus anginosus. After starting chemotherapy, a recurrent herpes simplex virus infection emerged as a significant complication. Studies showed that clinical reactivation of herpes simplex virus may easily occur in over 50% of patients receiving cytotoxic therapy [24]. In these cases, treatment options were topical and oral antiviral agents [25].

After 12 weeks of therapy with Dacarbazine and zoledronic acid, the patient presented with right mandibular necrosis. Bisphosphonate-related jaw osteonecrosis is a rare but very serious condition described in 1% to 10% of patients [26].

Nevertheless, radiotherapy can also cause osteonecrosis [27]. Osteoradionecrosis of the jaw is most frequently described in patients with head and neck cancers treated with curative or postoperative radiotherapy [28]. Our patient received both bisphosphonates and radiotherapy on the jaw bone. However, these two factors may be associated with an important trigger such as infection [29]. During chemotherapy, the patient was highly susceptible to any infection at the tumor site. However, the management of mandibular necrosis remains a significant clinical challenge, requiring local and systemic therapy, as well as surgery to remove the necrotic tissue.

## Conclusions

We presented a challenging case of a young 39-year-old woman, with primary amelanotic melanoma of the mandibular gingiva and tumor hyperprogressive disease. The histopathological diagnosis was challenging because of the similarities between leiomyosarcoma and amelanotic melanoma as well as GIST.

Even though it was an unfortunate and rare case of oral amelanotic melanoma of the jaw, with rapid progression on immunotherapy and several complications, including mandibular osteonecrosis, the patient had over 12 months of overall survival with metastatic disease and great performance status.

We also highlighted the importance of a multidisciplinary approach in this rare form of melanoma, which is the first reported clinical case of a female patient with oral AMM in Eastern Europe. As we encountered several obstacles along the therapeutic road, this clinical case presentation serves as a valuable tool for other oncology specialists or histopathology specialists who may find themselves facing a similarly complicated case.
